# Antihypertensive Effects of the Methanol Extract and the Ethyl Acetate Fraction from *Crinum zeylanicum* (Amaryllidaceae) Leaves in L-NAME-Treated Rat

**DOI:** 10.1155/2021/2656249

**Published:** 2021-07-03

**Authors:** Magloire Kanyou Ndjenda II, Elvine Pami Nguelefack-Mbuyo, Albert Donatien Atsamo, Christian Kuete Fofie, Chamberlin Fodem, Filomain Nguemo, Telesphore Benoit Nguelefack

**Affiliations:** ^1^Research Unit of Animal Physiology and Phytopharmacology, Faculty of Science, University of Dschang, P.O. Box 67, Dschang, Cameroon; ^2^Laboratory of Animal Physiology, Faculty of Science, University of Yaoundé I, P.O. Box 812, Yaounde, Cameroon; ^3^Institute of Neurophysiology, University of Cologne, Cologne 50931, Germany

## Abstract

Arterial hypertension (AHT) is a leading cardiovascular disease, with a high negative impact on the quality of life. *Crinum zeylanicum* (*C. zeylanicum*) leaves extract is used in the West region of Cameroon to treat AHT and heart problems. This study aimed to investigate the antihypertensive effect of *C. zeylanicum* extract in *N*^*ω*^-nitro-L‐arginine methyl ester- (L‐NAME-) induced hypertensive rats. The aqueous extract of *C. zeylanicum* (LAE) was obtained by lyophilizing the juice of triturated fresh leaves. The methanol extract (LME) prepared by maceration of the dried leaves was further partitioned to chloroform (LCF), ethyl acetate (LEAF), and residual (LRF) fractions. The total polyphenol, flavonoid content, and antiradical potentials of these extracts were determined. The curative antihypertensive and renal function protective effects of LME and LEAF were evaluated in vivo on L-NAME-induced hypertensive rats. Hypertension was induced in rats by oral administration of L-NAME (30 mg/kg/day) for 3 consecutive weeks. Thereafter, plant extracts were administered orally at the doses of 30, 60, and 120 mg/kg/day, concomitantly with L-NAME for three other weeks. Body weight, heart rate, and arterial blood pressure were measured at the end of each week throughout the experimental period. At the end of the treatment, 24-hour urine and plasma were collected to assay nitric oxide (NO), creatinine, and protein. The results revealed that LEAF has the higher content of total polyphenol and flavonoid and exhibited the best antiradical potential. Moreover, treatment of hypertensive rats with LME and LEAF significantly (*p* < 0.001) reduced AHT and heart rate. LME and LEAF significantly increased rat's body mass, plasmatic NO, and urinary creatinine and reduced urine NO and protein contents as compared to the L-NAME group. LME and its LEAF possess potent antihypertensive effects and further protect the renal function in L-NAME-induced hypertensive rats, thus supporting the use of *C. zeylanicum* in the management of AHT.

## 1. Introduction

The cardiovascular system, one of the most important systems of our body, is frequently disturbed by many disorders, including arterial hypertension (AHT). AHT is characterized by a persistent elevation of systolic/diastolic blood pressure equal to or greater than 140/90 mmHg [1]. It affects about 33.1% of patients in the world including 36.8% of males and 31.1% of females [2]. AHT is the primary risk factor for strokes and heart attacks as well as heart failure, renal impairment, and peripheral vascular disease, which contribute drastically to the morbidity of patients and the worsening of the quality of life.

AHT can be caused by endothelial dysfunction or any perturbation of the short- or long-term blood pressure regulation mechanism [3]. Experimentally, hypertension can be generated by many induction models including N^*ω*^-nitro-L-arginine methyl-ester (L-NAME). L-NAME is an L-arginine structural analog leading to the inhibition of the NO production and subsequently to endothelial dysfunction. It is well established that endothelium dysfunction is one of the primary characteristics of essential hypertension [4, 5], the most common form of arterial hypertension.

The treatment of AHT is very delicate because it is often resistant to existing drugs and in the majority of cases, many blood-pressure regulatory systems have to be targeted for proper achievement. Besides, the treatment must respect rigorously cardiovascular physiology and consider pathologies that may overlap like heart attack, heart failure, renal failure, and other organ complications. Among the panoply of existing drugs, very little is intended to address this issue, thus implying the practice of drug associations which unfortunately multiplies the side effects. It is, therefore, compulsory to seek new substances able to improve AHT and to repair subsequent damage and having very few side effects. The plant kingdom offers for this purpose many possibilities. Indeed, many classes of natural products from plant origin, led by polyphenol and its flavonoids subclass, have demonstrated good antihypertensive effects [6–8].

*C. zeylanicum* is a bulbous plant of the family Amaryllidaceae. The Amaryllidaceae is a monocotyledonous plant family, widely distributed around the globe and well known for its exceptional alkaloid principles and unique structural features, which have a diverse range of biological properties [9, 10]. The Amaryllidaceae includes about 80 genera and 1200 species distributed throughout the tropical and subtropical regions including warm temperate zones [10, 11]. Many plants from the genus *Crinum* have been shown to possess antihypertensive and cardiac moderating effects; they are also used in the treatment of several conditions such as headache, backache, swelling, hemorrhoids, wounds, and rheumatism [11–13]. Additionally, the fresh leaves of *C. zeylanicum* are chewed in the west region of Cameroon for the management of arterial hypertension and heart problems. This study evaluates the antihypertensive effects of *C. zeylanicum* extract and fractions on L-NAME-induced hypertensive rats.

## 2. Materials and Methods

### 2.1. Animal Material

The experiment was carried out on 72 male albino Wistar rats aged 11–13 weeks and weighing between 200 to 260 g. All the animals were fed with standard diet and water ad libitum in the animal house of the Research Unit of Animal Physiology and Phytopharmacology of Faculty of Science, University of Dschang, where they were exposed to a light/dark natural cycle (about 12/12 h). All experiments were carried out following the standard ethical guidelines for laboratory animal use and care (2010/63/EU).

### 2.2. Preparation of Plant Extracts

The leaves of *C. zeylanicum* were collected in BAZOU, a subdivision of Ndé division, west region of Cameroon, in June 2016 and identified at the National Herbarium of Cameroon in Yaoundé under the registration number 65654/HNC. The fresh leaves of *C. zeylanicum* were cut in pieces and ground. One kilogram of the obtained paste was pressed to extract the juice. The juice was filtered using hydrophilic cotton and Whatman filter paper (*n*°2). The resulting filtrate was freeze-dried to yield 300 g of powder. The other part (1 kg) was macerated for 72 h in methanol, filtered as for juice, and then evaporated at 65°C using a rotative evaporator. The methanol extract obtained was dried in a ventilated oven at 40°C for 24 h and weighed (248 g). One hundred grams of the methanol extract was suspended in distilled water and successively partitioned in chloroform (4 × 250 ml) and ethyl acetate (4 × 250 ml). For each passage, the aqueous suspension mixed with the extractive solvent was vigorously checked and stood for 20 minutes, and the organic phase was removed. Organic phases were concentrated to yield 22 g of chloroform fraction, 12.17 g of ethyl acetate fraction, and 62.83 g of the aqueous residue. The crude extracts and the fractions of the ethanol extract were stored in a hermetically closed vial at 4°C until used. They were analyzed for their contents in total polyphenols, flavonoids, and their ability to scavenge DPPH or nitric oxide. The most efficient crude extract together with the most active fraction was selected for in vivo hemodynamic effects.

### 2.3. Total Polyphenol Assay

Total polyphenol assay was performed using the Folin–Ciocalteu method as described by Singleton and Rossi [14]. Then, 250 *µ*l of each extract and fraction prepared at the concentration of 100 *µ*g/mL was mixed with 1.25 ml of Folin–Ciocalteu reagent and 1 ml of Na_2_CO_3_ at 7%. After shaking and incubation at 40°C for 30 min, the mixture absorbance was read with a spectrophotometer at 765 nm. The experiment was repeated 5 times. Results were expressed in mg equivalent of gallic acid/g of extract, referring to the gallic acid calibration curve.

### 2.4. Flavonoid Assay

Flavonoids assay was performed according to the method described previously [15]. Five hundred microliters (500 *µ*l) of each extract and fraction prepared in methanol at 500 *µ*g/ml was mixed with 1500 *µ*l of water and 150 *µ*l of NaNO_2_ at 5% was added; after 5 min of incubation at ambient temperature, 150 *µ*l of 10% AlCl_3_ was added and the mixture was incubated once more at ambient temperature 6 min. Then, 500 *µ*l of NaOH 4% was added, and after shaking, the volume was completed to 5 ml with distilled water and incubated for 5 min. The absorbance was then read with a spectrophotometer at 510 nm. The experiment was repeated 5 times and results were expressed in mg equivalent of quercetin/g of extract, referring to the quercetin calibration curve.

### 2.5. DPPH Radical Scavenging Test

The radical scavenging potential of the extracts was evaluated using DPPH solution as previously described by Nguelefack-Mbuyo et al. [16]. Ascorbic acid was used as a reference. Briefly, 150 *µ*l of varying concentration (1–300 *μ*g/ml) of extracts, fractions, or ascorbic acid (used in this study as a reference compound) was added to 850 *µ*l of methanol and the absorbance read at 517 nm against a blank made up of methanol (*A*_1_). Then 500 *µ*l of DPPH (0.063 mg/ml in methanol) was added to the medium, the whole was incubated in the dark for 20 min, and the absorbance (*A*_2_) was measured spectrophotometrically at 517 nm. The experiment was done in triplicate and the antioxidant activity (%) was calculated using the following equation: antioxidant activity = ((*A*_2_–*A*_1_)_control_−(*A*_2_−*A*_1_)_sample_) × 100/(*A*_2_−*A*_1_)_control_. Control tubes were those containing DPPH without any antioxidant substance. The EC50 of each tested substance was calculated using GraphPad Prism 5.01.

### 2.6. Nitric Oxide (NO) Production Assay

In vitro, NO generated from sodium nitroprusside at physiological pH interacts with oxygen to produce nitrite ions that can be measured by Griess reaction [17]. A sodium nitroprusside solution (10 mM) was freshly prepared in 0.1 mM phosphate buffer (pH 7.4) and1520 *µ*l of it was introduced in test tubes in presence of 180 *µ*l of distilled water or test substances (plant extracts, fractions, or ascorbic acid) at the concentrations range of 1–300 *µ*g/ml. The mixture was incubated at 25°C for 2 h 30 min. Then 500 *µ*l of the incubated mixture was removed for nitrite measurement using Griess reaction. Briefly, 500 *µ*l of each experimental sample was incubated for 5 min with 500 *µ*l 1% sulphanilamide prepared in 5% phosphoric acid. Then, 500 *µ*l of 0.1% naphthyl ethylenediamine in distilled water was added into the reaction milieu and incubated for another 5 min. The absorbance of the chromophore formed was read at 530 nm. The quantity of nitrite in each sample was determined using the standard sodium nitrite curve.

### 2.7. Blood Pressure and Heart Rate Recording

For the in vivo assessment of the effect of *C. zeylanicum* on blood pressure and heart rate, the crude methanol extract (LME) and its ethyl acetate fraction (LEAF) were selected. They were chosen according to their content in polyphenols and flavonoids and their ability to inhibit DPPH radical and to increase nitric oxide production.

Seventy-two male albino Wistar rats divided into 9 groups of 8 animals each were treated as follows: Group 1, naive: animals received distilled water during the six weeks of treatment; Group 2, disease control: animals received L-NAME and distilled water during the treatment period; Group 3, animals received L-NAME alone for 3 weeks and then L-NAME + captopril (20 mg/kg/day, reference drug) for 3 additional consecutive weeks; the remaining six groups received L-NAME alone for 3 weeks and thereafter L-NAME + LME (groups 4, 5, and 6) or LEAF (groups 7, 8, and 9) at respective doses of 30, 60, and 120 mg/kg. The dose of 60 mg/kg was obtained as therapeutic dose following the traditional healer indications. This dose was further divided or multiplied by 2 to obtain 30 mg/kg and 120 mg/kg, respectively.

L-NAME was administered orally at the dose of 30 mg/kg/day. During the six-week treatment period, heart rate and arterial blood pressure were weekly measured using the noninvasive tail-cuff method. To record these parameters, each rat was placed in a retention cage at a temperature of 32°C for 15 minutes to facilitate detection of the pulse in the caudal artery of the animal. Then, an inflatable photoelectric sensor was placed around the tail of the rat. The automatic swelling of the sensor exerted pressure on the caudal artery progressively until complete occlusion of the artery. The blood circulation in the artery was gradually restored by automatic deflation of the sensor. Data were collected by IITC Life Science Data Acquisition software for MRBP tail-cuff blood pressure coupled to a computer. The data (systolic blood pressure, diastolic blood pressure, and heart rate) were displayed on the computer screen.

At the end of the treatment, 24-hour urine was collected and animals were then anesthetized by intraperitoneal injection of thiopental (50 mg/kg). Blood, heart, kidney, and aorta were collected. Plasma was separated by centrifugation at 3000 rpm for 15 minutes. Organs were weighed. Collected urine and plasma were used to determine the concentration of nitric oxide, creatinine, and protein in each sample. The glomerular filtration rate (GFR) was calculated using the Jaffe method.

### 2.8. Biochemical Analysis

Plasma and urine nitric oxide contains were measured using Griess reagent. For the assay, 350 *µ*l of 1% sulphanilamide was mixed with 350 *µ*l of plasma or urine. After 5 minutes of incubation, 350 *µ*l of 0.1% naphthyl ethylenediamine was added, additional 5 minutes incubation was observed, and the absorption of the chromophore was read at 546 nm.

The quantification of plasma protein was done as described by Pessoa et al. [18]: 250 *µ*l plasma was mixed with 1 mL of Biuret reagent. The mixture was incubated for 30 min and the absorbance was read at 540 nm. Urinary protein assay was done by the Bradford method: 100 *µ*l of Bradford reagent was added to 50 *µ*l of urine and after 30 min incubation, the mixture absorbance was read at 540 nm using a microplate reader.

The kinetic method was used for creatinine assay both in plasma and in urine: 200 *µ*l of plasma or urine (diluted at 1/10) was mixed with 500 *µ*l of NaOH (3.3 mM) and 500 *µ*l of picric acid (8.13 mM). The absorbance of the mixture was measured after 1 and 3 min at 500 nm. A standard curve of creatinine was constructed and used to determine creatinine levels in various samples. Creatinine clearance was used as an estimation of the glomerular filtration rate (GFR) and was calculated as follows: GFR = *U *×* V*/*P *×* *1440, where *U* is urine creatinine, *V* is 24 hours' urine volume, *P* is plasma creatinine, and 1440 is time in seconds corresponding to 24 hours.

### 2.9. Statistical Analysis

Data are expressed as mean ± SEM. One-way ANOVA with Tukey posttest were used to compare parameters with one dependent variable while two-way ANOVA repeated measure with Bonferroni posttest was used for data with two dependent variables. In the in vitro studies, efficient concentrations 50 (EC50) were automatically deducted from the nonlinear regression curve (log agonist–response). GraphPad Prism version 5.03 software was used for data analysis. Means were considered significantly different at *p* < 0.05.

## 3. Results

### 3.1. Total Polyphenols and Flavonoids Content in the Leaves of *Crinum zeylanicum*

**Figure 1:**
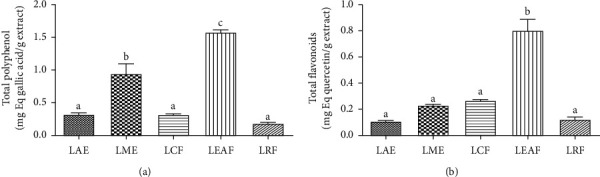
Total polyphenols and flavonoids content of *Crinum zeylanicum* extracts and fractions. The ethyl acetate fraction (LEAF) presented the highest content in polyphenols and flavonoids. *N* = 5 replications. Bars with different letters are significantly different at least at *p* < 0.05. Analysis was done with ANOVA one-way followed by Tukey posttest. LAE: leaves aqueous extract, LME: leaves methanol extract, LCF: leaves chloroform fraction, LEAF: leaves ethyl acetate fraction, and LRF: leaves residual fraction.

As depicted in Figure 1(a), the total polyphenols content was significantly higher in LME (0.94 ± 0.14 mg equivalent gallic acid/g extract) than in LAE (0.31 ± 0.01 mg equivalent gallic acid/g extract). The fractionation of LME concentrated polyphenols in the LEAF which was significantly richer (1.57 ± 0.03 mg equivalent gallic acid/g extract) than the crude LME.

Concordantly, LME was more concentrated in flavonoids (0.22 ± 0.00 mg equivalent quercetin/g extract) than the LAE (0.10 ± 0.00 mg equivalent quercetin/g extract). Also, LEAF flavonoid content (0.80 ± 0.08) was significantly higher, compared to that of LCF (0.31 ± 0.00) and RCF (0.17 ± 0.00). A significant difference was observed only between the flavonoids content of LEAF and all other extracts and fractions (Figure 1(b)).

### 3.2. Effect of *Crinum zeylanicum* Leaves Extracts on DPPH Radical and NO Production

**Figure 2:**
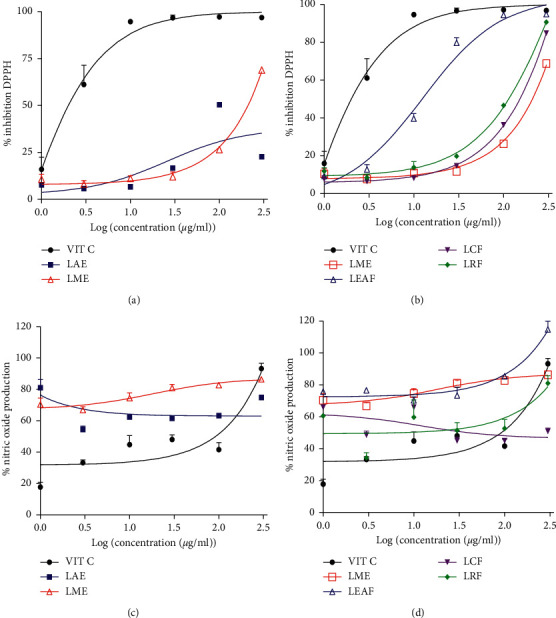
*Crinum zeylanicum* leaves extracts and fractions exhibited DPPH radical scavenging (a, b) effects and increased the in vitro nitric oxide production (c, d). Analysis was done using a nonlinear regression curve. LAE: leaves aqueous extract, LME: leaves methanol extract, LCF: leaves chloroform fraction, LRF: leaves residual fraction, and VIT C: vitamin C (reference drug).

Extracts and fractions from the leaves of *C. zeylanicum* induced concentration-dependent DPPH scavenging activities. Although LME induced the highest maximal activity (55.16 ± 16.19%) as compared to LAE, the effects of both crude extracts were not statistically different (Figure 2(a)). The fractions of LME were more potent than the crude extract. The LEAF exhibited the best activity with an EC_50_ of 12.61 *µ*g/ml as compared to the LCF (1320.00 *µ*g/ml) and the LRF (475.10 *µ*g/ml). Nevertheless, LEAF was 27-fold less efficient than the vitamin C (EC_50_: 0.47) used as a reference substance (Figure 2(b)).

As depicted in Figures 2(c) and 2(d), *C. zeylanicum* extracts and fractions increased the production of NO with percentages ranging from 20 to 120%. LEAF also exhibited the best activity.

### 3.3. Effect of the Methanol Extract and Ethyl Acetate Fraction from the Leaves of *Crinum zeylanicum* on the Systolic Blood Pressure

**Figure 3:**
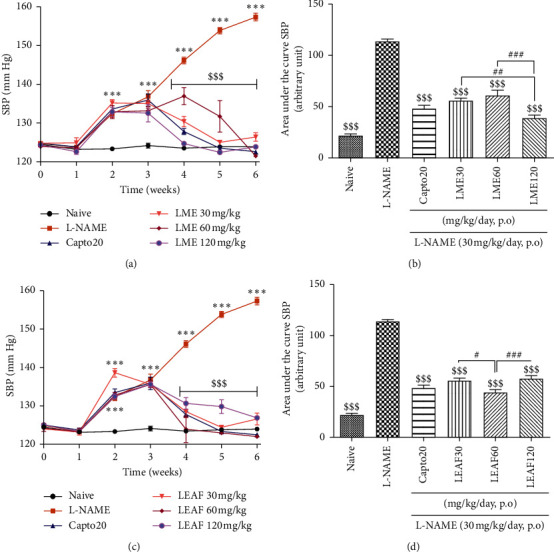
Repeated administration of the methanol extract and the ethyl acetate fraction from the leaves of *Crinum zeylanicum* time-dependently reduced systolic blood pressure in rats treated with L-NAME; 6 ≤ *n* ≤ 8. Analysis was done with ANOVA two-way followed by Bonferroni posttest (panels A and C) or ANOVA one-way followed by Tukey posttest (panels B and D). ^*∗*^*p* < 0.05, ^*∗∗*^*p* < 0.01, and ^*∗∗∗*^*p* < 0.001: significant difference as compared to the naive group. ^$$$^*p* < 0.001: significant difference as compared to L-NAME treated group. ^#^*p* < 0.05, ^##^*p* < 0.01, and ^###^*p* < 0.001: significant difference between concerned groups. Capto20: captopril, LME: leaves methanol extract, LEAF: leaves ethyl acetate fraction, SBP: systolic blood pressure, and naive: normal untreated animals.

Oral administration of L-NAME at the dose of 30 mg/kg/day induced a progressive increase in rat systolic blood pressure that was significantly (*p* < 0.001) high from the second week of the experiment as compared to the naive group. The adjunction of LME and LEAF at the doses of 30, 60, and 120 mg/kg/day progressively and significantly (*p* < 0.001) reduced the systolic blood pressure. The treatment was time-dependently significant (*p* < 0.001) (Figures 3(a) and 3(c)). The area under the curve was calculated to estimate the overall effect of treatment and to allow comparing the effect of different doses. With the LME, the dose of 120 mg/kg/day was significantly more effective than lower doses. The dose 60 mg/kg/day of the LEAF showed the best activity with a significant difference as compared to 30 mg/kg/day (*p* < 0.05) and 120 mg/kg/day (*p* < 0.001) (Figures 3(b) and 3(d)). No significant difference was observed between plant extracts and captopril, neither between both plant extracts at corresponding doses.

Thirty percent of death was observed in L-NAME control group. This percentage was reduced to 25% in captopril and LEAF 30 mg/kg groups and 12.5% in LEAF 60 and 120 mg/kg while no death was observed in naive and groups treated with LME at 30 and 60 mg/kg.

### 3.4. Effect of the Methanol Extract and Ethyl Acetate Fraction from the Leaves of *Crinum zeylanicum* on the Diastolic Blood Pressure

**Figure 4:**
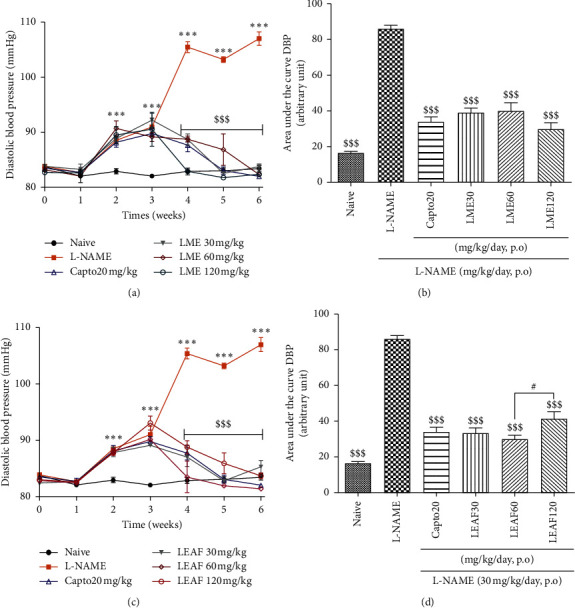
Repeated administration of the methanol extract and the ethyl acetate fraction from the leaves of *Crinum zeylanicum* time-dependently reduced diastolic blood pressure in rats treated with L-NAME; 6 ≤ *n* ≤ 8. Analysis was done with ANOVA two-way followed by Bonferroni posttest (a, c) or ANOVA one-way followed by Tukey posttest (b, d). ^*∗*^*p* < 0.05, ^*∗∗*^*p* < 0.01, and ^*∗∗∗*^*p* < 0.001: significant difference as compared to the naive group. ^$$$^*p* < 0.001: significant difference as compared to L-NAME treated group. ^#^*p* < 0.05: significant difference between concerned groups. Capto20: captopril, LME: leaves methanol extract, LEAF: leaves ethyl acetate fraction, DBP: diastolic blood pressure, and naive: normal untreated animals.

Daily oral administration of L-NAME significantly (*p* < 0.001) increased diastolic blood pressure which reaches 107.66 ± 1.26 mmHg compared to the naive group (82.08 ± 0.39 mmHg). Oral administration of both LME (Figures 4(a) and 4(b)) and LEAF (Figures 4(c) and 4(d)) at all doses (30, 60, and 120 mg/kg) significantly (*p* < 0.001) reduced diastolic blood pressure in L-NAME treated animals. LEAF at 60 mg/kg and LME at 120 mg/kg were the most effective doses. They reduced the parameter to 81.42 ± 0.15 and 82.40 ± 0.28 mmHg, respectively.

### 3.5. Effect of the Methanol Extract and Ethyl Acetate Fraction from the Leaves of *Crinum zeylanicum* on the Heart Rate

**Figure 5:**
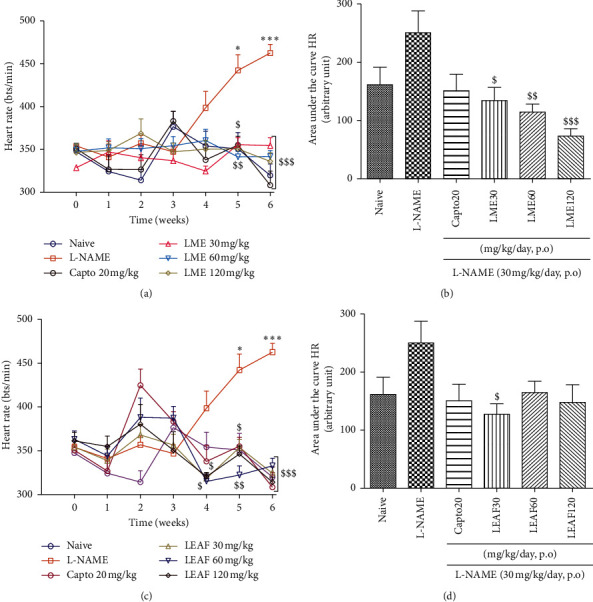
Repeated administration of the methanol extract and the ethyl acetate fraction from the leaves of *Crinum zeylanicum* prevented the rise in heart rate induced by L-NAME; 6 ≤ *n* ≤ 8. Analysis was done with ANOVA two-way followed by Bonferroni posttest (a, c) or ANOVA one-way followed by Tukey posttest (b, d). ^*∗*^*p* < 0.05, ^*∗∗*^*p* < 0.01, and ^*∗∗∗*^*p* < 0.001: significant difference as compared to the water-treated group. ^$^*p* < 0.05, ^$$^*p* < 0.01, and ^$$$^*p* < 0.001: significant difference as compared to L-NAME treated group. Capto20: captopril, LME: leaves methanol extract, LEAF: leaves ethyl acetate fraction, HR: heart rate. Naïve: normal untreated animals.

None of the treatments significantly affected the heart rate during the first three weeks of the experiment. It was only from the fourth week that L-NAME progressively increased the heart rate to 462.66 ± 10.28 bt/min at the 6^th^ week. LME administration significantly and dose-dependently decreased heart rate as compared to the L-Name group. Although a point-by-point analysis showed a significant bradycardia effect of LEAF as from the fourth week of the experiment, the global analysis using area under the curve indicates that only the dose of 30 mg/kg was significantly (*p* < 0.05) effective (Figure 5).

### 3.6. Effect of *Crinum zeylanicum* Extracts on the Body and Target Organs Masses

The effects of *C. zeylanicum* extracts on rat body mass as well as target organs masses are shown in Table 1. These results show that the repeated administration of L-NAME resulted in a significant (*p* < 0.001) reduction in rat body mass (−49.21 ± 5.05 g) compared to the naive group (13.33 ± 4.80 g). LME at 30 mg/kg (39.00 ± 4.86 g) and LEAF at 30 mg/kg (80.00 ± 4.38 g) and 60 mg/kg (35.76 ± 4.14 g) significantly (*p* < 0.001) reduced this loss in rats' body mass as compared to L-NAME treated group. Only LEAF at 30 mg/kg significantly (*p* < 0.05) decreased the relative aorta mass, as compared to both naive and L-NAME treated groups. None of the treatments significantly affected the heart and kidney relative weights after the six weeks of treatment, when compared to the L-NAME group.

### 3.7. Effect of the Methanol Extract and the Ethyl Acetate Fraction from the Leaves of *Crinum zeylanicum* on the Renal Function

Table 2 presents data of the effects of LME and LEAF on the diuresis, plasma, and urine contents in NO, proteins, and creatinine, as well as on the glomerular filtration rate in L-NAME treated animals.

The oral administration of L-NAME did not significantly affect the urinary output. Although none of the plant extracts significantly affected the parameter, LME tends to increase it while LEAF leans towards a reduction.

L-NAME administration did not significantly affect the concentration of NO in the plasma, as compared to the naive group. *C. zeylanicum* extracts tend to increase it. However, L-NAME treatment significantly (*p* < 0.001) increased the urinary excretion of NO. Captopril and both plant extracts significantly (*p* < 0.001) completely reversed the effect of L-NAME.

Repeated oral administration of L-NAME significantly (*p* < 0.05) increased the plasma protein content. This was significantly reversed by the coadministration of LME at all the doses used and LEAF at the dose of 120 mg/kg. Concerning the urinary excretion of proteins, L-NAME did not significantly affect the parameter. However, LME and LEAF significantly reduced the urinary protein contents as compared to both naive and L-NAME treated groups.

The different treatments did not significantly affect plasma creatinine content. But L-NAME administration drastically (*p* < 0.001) reduced the urinary excretion of creatinine, as compared to the naive group. Captopril and the *C. zeylanicum* extracts as well significantly reversed the effect of L-NAME by significantly increasing the creatinine content in animal urine.

The creatinine clearance was used to estimate the glomerular filtration rate. As shown in Table 2, treatment with L-NAME significantly (*p* < 0.05) reduced the glomerular filtration rate by 73% compared to the naive group. Captopril and the plant extracts reversed the L-NAME effect but only captopril (*p* < 0.05) and LME (*p* < 0.001) significantly increased the glomerular filtration rate as compared to L-NAME treated group.

## 4. Discussion

Arterial hypertension is one of the leading cardiovascular disorders, with more than 1 billion patients affected all over the world. Endothelial dysfunction characterized by the reduction in NO biodisponibility is the common cause of essential hypertension, which represents at least 90% of hypertensive cases [19]. L-NAME-induced arterial hypertension mimics perfectly the clinical conditions of essential hypertension and has been intensively used as an experimental model in drug discovery against arterial hypertension and its complications [20].

In the present study, the effects of the methanol extract of the leaves of *C. zeylanicum* and its ethyl acetate fraction on arterial hypertension and the renal function were evaluated in L-NAME treated rats. It has been extensively shown that polyphenols, especially flavonoid compounds, possess antihypertensive effects and that this activity is closely related to their antioxidant capacities [21–23]. Based on this knowledge, we hypothesized that *C. zeylanicum* extracts may induce their claimed antihypertensive effects thanks to their content in polyphenols and flavonoids. For this reason, the study started with the quantification of these substances in the crude extracts and their fractions. The purpose of the fractionation was to concentrate as much as possible this class/subclass of compounds in one fraction. The methanol extract was richer in both polyphenols and flavonoids than the aqueous extract. The LEAF showed a higher content as compared to either crude extracts or other fractions of the LME. Concordantly, the LEAF and LME exhibited the best radical scavenging effects, suggesting that their antiradical effect could be related to their content in polyphenols and flavonoids.

Surprisingly, LME and LEAF instead increase the NO production. Although this result seems contradictory to the antioxidant activity, it might be rather interesting as far as the antihypertensive effect is concerned. In fact, NO is an endothelial-derived factor with a potent vasorelaxant effect [24]. Increasing its content in vivo will lead to a vasodilation, resulting in a reduction in peripheral resistance and, therefore, a reduction in arterial hypertension [24]. Many nitric derivatives or nitric oxide donors are widely used in the management of cardiovascular diseases including arterial hypertension [25]. In this line, we hypothesized that LME and LEAF could possess good antihypertensive effects in a model with endothelial deficiency. The curative effect of both extracts was then evaluated in L-NAME-induced hypertensive rats.

LME and LEAF administered orally on a daily basis not only significantly antagonized the progressive increase in blood pressure induced by the L-NAME, but also reduced the already elevated values of the blood pressure to the normal values, demonstrating a potent antihypertensive activity. Both extracts also significantly reduced the elevated heart rate induced by L-NAME. These findings suggest that the bradycardia effects of these extracts may contribute to their antihypertensive effect. Besides, it seems difficult to consider the NO pathway in these effects since both extracts were unable to significantly increase the plasma concentration of this cell signaling mediator.

To assess the effect of *C. zeylanicum* extracts on renal function, we evaluated several parameters including diuresis and NO, proteins, and creatinine contents in both the plasma and urine. LEAF significantly reduced the urine output while LME tends to increase it. This result suggests the differential activity of the two extracts, which might be related to a difference in their chemical composition, as observed in the quantitative phytochemical analysis. The reduction in diuresis observed with LEAF may negatively impact its antihypertensive effect. Similar to captopril, both plant extracts significantly reduced the urinary excretion of proteins but increased the urinary excretion of creatinine and the glomerular filtration rate (GFR). The GFR is the optimal way to measure kidney function and, when combined with proteinuria or albuminuria, can help determine the extent of chronic kidney diseases [26] (Gounden et al., 2020). The fact that *C. zeylanicum* extracts were able to reduce the proteinuria and increase the creatinuria and the GFR denotes its ability to protect the kidney against chronic kidney diseases which is a well-known kidney alteration in the L-NAME model [27].

The most effective dose on blood pressure for LME was 120 mg/kg while that of LEAF was 60 mg/kg. Similar effect was also observed on heart rate and glomerular filtration rate where LME at 120 mg/kg showed the best activity. However, considering the total activities evaluated, no marked difference was observed between the effects of the two plant extracts. Treatment with plan extracts reduced the percentages of mortality in L-NAME treated animals. These results suggest a relatively nontoxic effects of the plant extracts at the doses used. Nevertheless, toxicological studies are requested to ascertain this hypothesis.

## 5. Conclusion

In this study, we examined the polyphenols and flavonoids contents as well as the antihypertensive effects of extracts from the leaves of *C. zeylanicum*. We found that LEAF has the higher content of total polyphenol and flavonoid and exhibited the best antiradical potential. LME and LEAF significantly reduced AHT and heart rate. LME and LEAF significantly increased plasmatic NO and urinary creatinine and reduced urine NO and protein contents.

Taking all together, it can be concluded that the methanol extract and the ethyl acetate fraction from the leaves of *C. zeylanicum* have anti-hypertensive effect with no significant difference between the two extracts. The antioxidant and cardiac moderating effects might contribute to this anti-hypertensive activity although other regulating effects such as reduction in peripheral resistances cannot be ruled out. *C. zeylanicum* extracts might protect against renal function alteration in the condition of arterial hypertension. These data support the use of *C. zeylanicum* in the treatment of arterial hypertension.

## Figures and Tables

**Table 1:** Effect of chronic administration of captopril, methanol extracts, and ethyl acetate fraction obtained from leaves of *Crinum zeylanicum* on the gain in body weight and relative weight of aorta, heart, and kidney in L-NAME treated rats.

Parameters	Treatments								
	Naive	L-NAME	L-NAME + capto20	L-NAME + LME 30	L-NAME + LME 60	L-NAME + LME 120	L-NAME + LEAF 30	L-NAME + LEAF 60	L-NAME + LEAF 120
Gain in body weight (g)	13.33 ± 4.8^$$$^	−49.21 ± 5.05	−65.00 ± 6.34	39.71 ± 4.86^$$$^	−45.40 ± 3.11	−7.95 ± 1.45^$$$^	80.04 ± 5.38^$$$^	35.76 ± 4.14^$$$^	−6.50 ± 0.80^$$$^
Aorta relative weight (%)	0.30 ± 0.03	0.24 ± 0.02	0.15 ± 0.01	0.19 ± 0.01	0.21 ± 0.02	0.19 ± 0.01	0.14 ± 0.01^$^	0.23 ± 0.02	0.20 ± 0.01
Heart relative weight (%)	2.33 ± 0.09	1.98 ± 0.13	1.98 ± 0.13	2.08 ± 0.06	2.09 ± 0.06	2.12 ± 0.03	1.98 ± 0.13	2.02 ± 0.08	2.29 ± 0.05
Kidney relative weight (%)	2.32 ± 0.06	1.78 ± 0.07	2.03 ± 0.12	1.98 ± 0.04	2.04 ± 0.09	1.95 ± 0.04	1.04 ± 0.30	1.11 ± 0.30	0.95 ± 0.25

L-NAME: N^*ω*^-nitro-L-arginine methyl-ester, LME: leaf methanol extract, LEAF: leaf ethyl acetate fraction, capto: captopril, and naive: normal untreated animals. The number associated with the administered substances indicates the oral dose in mg/kg/day. 6 ≤ *n* ≤ 8. Analysis was done with ANOVA one-way followed by Tukey posttest. ^$^*p* < 0.05, ^$$^*p* < 0.01, and ^$$$^*p* < 0.001: significant difference as compared to L-NAME treated group.

**Table 2:** Effect of chronic administration of captopril, methanol extract, and ethyl acetate fraction obtained from leaves of *Crinum zeylanicum* on diuresis, glomerular filtration rate, plasma and urine nitric oxide, proteins, and creatinine in L-NAME treated rats.

Parameters		Treatments								
		Naive	L-NAME	L-NAME + capto20	L-NAME + LME 30	L-NAME + LME 60	L-NAME + LME 120	L-NAME + LEAF 30	L-NAME + LEAF 60	L-NAME + LEAF 120
Diuresis (ml/kg/h)		1.07 ± 0.11	1.26 ± 0.08	1.43 ± 0.23	1.70 ± 0.17	1.71 ± 0.23	1.77 ± 0.30	0.810 ± 0.186	0.68 ± 0.09	1.59 ± 0.18
NO (*µ*g/*µ*l)	Plasma	4.35 ± 0.70	4.41 ± 0.31	5.80 ± 0.37	3.20 ± 0.39	5.06 ± 0.37	4.58 ± 0.31	5.70 ± 0.33	4.78 ± 0.26	5.35 ± 0.53
	Urine	1.99 ± 0.27^$$$^	5.05 ± 0.49	2.57 ± 0.92^$$^	2.31 ± 0.35^$$$^	0.60 ± 0.05^$$$^	0.54 ± 0.05^$$$^	0.64 ± 0.06^$$$^	0.53 ± 0.10^$$$^	0.42 ± 0.04^$$$^
Proteins (*µ*g/ml)	Plasma	6.01 ± 0.63^$^	9.54 ± 0.44	7.25 ± 0.40	5.62 ± 1.01^$$^	4.05 ± 0.48^$$$^	4.33 ± 0.98^$$$^	6.73 ± 1.03	7.52 ± 1.21	3.40 ± 1.13^$$$^
	Urine	9.59 ± 0.35	9.85 ± 1.11	5.92 ± 0.251^$^	5.30 ± 0.77^$$^	6.33 ± 0.98^$^	8.61 ± 0.89	5.90 ± 0.88^$$^	6.46 ± 0.65^$^	5.26 ± 0.58^$$$^
Creatinine (mg/ml)	Plasma	3.66 ± 0.48	2.85 ± 0.55	2.60 ± 0.26	2.47 ± 0.20	2.42 ± 0.41	2.38 ± 0.33	2.66 ± 0.34	2.88 ± 0.49	4.33 ± 0.52
	Urine	3.47 ± 0.44^$$$^	1.02 ± 0.04	3.20 ± 0.55^$$$^	1.14 ± 0.16	1.49 ± 0.14	6.06 ± 0.37^$$$^	1.16 ± 0.19	5.11 ± 0.50^$$$^	2.19 ± 0.36^$$$^
GFR (*µ*L/min)		0.37 ± 0.06^$^	0.10 ± 0.01	0.35 ± 0.05^$^	0.20 ± 0.04	0.24 ± 0.03	1.01 ± 0.16^$$$^	0.10 ± 0.02	0.31 ± 0.07	0.23 ± 0.05

L-NAME: N^*ω*^-nitro-L-arginine methyl-ester, LME: leaf methanol extract, LEAF: leaf ethyl acetate fraction, capto20: captopril, and naive: normal untreated animals. The number associated with the administered substances indicates the oral dose in mg/kg/day. 6 ≤ *n* ≤ 8. Analysis was done with ANOVA one-way followed by Tukey posttest. ^$^*p* < 0.05, ^$$^*p* < 0.01, and ^$$$^*p* < 0.001: significant difference as compared to L-NAME treated group.

## Data Availability

The data used and analyzed in this study are available from the corresponding author on reasonable request.
